# A realistic approach to evaluating the effect of baseline lipid profile in postcoronary artery bypass grafting surgery

**DOI:** 10.1002/clc.24132

**Published:** 2023-08-18

**Authors:** Ali Sheikhy, Aida Fallahzadeh, Saeed Sadeghian, Mina Pashang, Abbas Ali Karimi, Jamshid Bagheri, Hossein Ahmadi‐Tafti, Kaveh Hosseini

**Affiliations:** ^1^ Cardiac Primary Prevention Research Center, Cardiovascular Diseases Research Institute Tehran University of Medical Sciences Tehran Iran; ^2^ Tehran Heart Center, Cardiovascular Diseases Research Institute Tehran University of Medical Sciences Tehran Iran; ^3^ Non‐Communicable Disease Research Center, Endocrinology and Metabolism Population Sciences Institute Tehran University of Medical Sciences Tehran Iran

**Keywords:** Coronary artery bypass grafting (CABG), HDL, LDL, lipid profile, remnant cholesterol, Restricted Cubic Splines (RCS)

## Abstract

**Background:**

There are still many uncertainties in the association between lipid profile and postcoronary artery bypass grafting (CABG) outcomes. Although simplifying the association to linear equations makes it understandable but cannot explain many findings.

**Hypothesis:**

There is a nonlinear associatin between lipid profile indices and adverse outcomes after CABG.

**Methods:**

A total of 17 555 patients who underwent isolated CABG between 2005 and 2016 were evaluated. During the median follow‐up of 75.24 months, the Restricted Cubic Splines (RCS) estimated from the Cox regression model adjusted for all possible confounders was applied to show a nonlinear relationship of lipid profile contents with the “ln hazard ratio” of mortality and major cerebro‐cardiac events (MACCE).

**Results:**

The relationship between LDL‐C and HDL‐C with all‐cause mortality was nonlinear (nonlinear *p* were .004 and <.001, respectively). The relationship between remnant cholesterol and all‐cause mortality was linear (linearity *p* = .023). Among men, those in the highest LDL‐C level (Q4, LDL‐C > 114) and those in the lowest HDL‐C level (Q1, HDL‐C < 30) showed a significantly higher risk of all‐cause mortality compared to other groups (compared with Q3, LDL‐C Q4, HR = 1.16, 95% confidence interval [CI]:1.02–1.26, *p* = .014; HDL‐C Q1, HR = 1.14, 95% CI: 1.01–1.31, *p* = .041). Female patients in the lowest HDL‐C level (Q1, HDL‐C < 30) showed a significantly higher (compared with Q3, HR = 1.14, 95% CI:1.01–1.31, *p* = .028) and those in the highest HDL‐C level (Q4, HDL‐C > 43) showed a significantly lower (compared with Q3, HR = 0.74, 95% CI:0.58–0.98, *p* = .019) risk of all‐cause mortality.

**Conclusion:**

Determining a universal cut off for components of lipid profile may be misleading and should better be revised. Extreme values (very low or very high) for HDL‐C and LDL‐C have different effects on cardiovascular outcomes.

## Graphical abstract

There are still many uncertainties in the association between lipid profile and post coronary artery bypass grafting (CABG) outcomes. Although simplifying the association to linear equations makes it understandable but cannot explain many findings. A total of 17 555 patients who underwent isolated CABG were evaluated. The Restricted Cubic Splines (RCS) estimated from the Cox regression model adjusted for all possible confounders was applied to show a nonlinear relationship of lipid profile contents with the “ln hazard ratio” of mortality and major cerebro‐cardiac events (MACCE). The relationship between LDL‐C and HDL‐C with all‐cause mortality was nonlinear (nonlinear *p* were .004 and <.001, respectively). The relationship between remnant cholesterol and all‐cause mortality was linear (linearity *p* = .023).

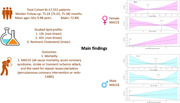

## 1 INTRODUCTION

Previous studies have established the role of high levels of low‐density lipoprotein cholesterol (LDL‐C) and low levels of high‐density lipoprotein cholesterol (HDL‐C) as risk factors in the development and progression of atherosclerotic cardiovascular disease (ASCVD).^1^, ^2^ LDL is an ApoB‐containing lipoprotein which can cross the endothelial barrier, especially in the presence of endothelial dysfunction, and become trapped and initiate the formation of lipid‐rich atheromatous plaque.^2^ HDL‐C is known to have athero‐protective effects due to reverse cholesterol transport and anti‐inflammatory properties.^3^ Additionally, there is a very atherogenic lipoprotein known as remnant cholesterol (all plasma cholesterol that is not LDL‐C or HDL‐C) which is composed of triglyceride‐rich remnant lipoproteins.^4^ Remnant cholesterol, similar to LDL‐C, can potentially infiltrate the arterial intima, and cause atherosclerosis due to its cholesterol content,^5^ while triglycerides are unlikely to cause atherosclerosis on their own.^6^ It has been shown that high levels of remnant cholesterol are predictive of coronary artery disease (CAD), especially in those with normal total cholesterol levels.^7^, ^8^

Numerous clinical trials have demonstrated that lipid lowering therapy improves adverse outcomes in patients with established CAD.^9^, ^10^, ^11^ However, a significant residual risk of atherosclerosis complications remains in patients having target low lipid profile.^12^, ^13^ Most of the previous studies considered lipid indices as continuous variables for linear correlation or divided lipid indices into categorical variables according to the existing guidelines and percentage scales.^14^, ^15^, ^16^ However, considering a linear relationship between lipid indices and adverse outcomes, cannot explain many findings. Limited data are available on the nonlinear relationship between lipids, especially the effect of low LDL‐C and high HDL‐C and adverse outcomes.^17^, ^18^, ^19^, ^20^, ^21^

Although the importance of HDL‐C and LDL‐C as independent predictors of atherosclerosis has been reported, their role in the prediction of survival and adverse outcome after coronary artery bypass grafting (CABG) is less certain. Moreover, there is little evidence regarding the pattern of association. Therefore, this study aimed to evaluate the association of LDL‐C, HDL‐C, and remnant cholesterol with post CABG outcomes and depict the pattern of this association.

## 2 MATERIAL AND METHOD

### 2.1 Data source and study population

This is a registry‐based prospective study conducted in Tehran Heart Center (THC) clinical registry, which includes patients with CAD who underwent isolated CABG between 2005 and 2016. This conducted study approved by the Tehran Heart Center ethical board (IR‐THC‐13799). Presented study didn't meet the criteria for informed consent whereas individuals name kept anonymous excepting for corresponding author and data base chief, consequently “informed consent waiver” was obtained from the THC ethical committee. Involving patients' data was in agreement with standards of Declaration of Helsinki. We assessed all patients who underwent isolated CABG (24 328 patients), and patients with inadequate data were excluded from the current study. The more specific data regarding the missing variables and exclusion criteria were reported in Supporting Information.

### 2.2 Follow‐up protocol

The patients were followed at 4 or 6 and 12 months after the procedure and yearly via direct visits. Patients that were incapable to attend direct clinic visits were followed through telephone interviews. The patients' demographic characteristics, CAD risk factors (i.e., diabetes [DM], hypertension [HTN], dyslipidemia [DLP], case history of CAD, cigarette smoking [CS], opium consumption, and obesity), laboratory results (hemoglobin, creatinine, HDL‐C, LDL‐C, and Total Cholesterol), history of previous disease, ejection fraction, number of grafts, and occurrence of major adverse cardiocerebrovascular events (MACCEs) were recorded. Complete definitions of all variables were also mentioned within the Supporting Information.

### 2.3 Study endpoints and variables

The primary endpoints of this study were all‐cause mortality and occurrence of MACCEs (defined as composite of death, myocardial infarction, stroke, or transient ischemic attack [TIA], and a need for repeat revascularization [percutaneous coronary intervention or redo‐CABG]). The remnant cholesterol was measured by taking the value of total cholesterol minus the HDL and LDL values.

### 2.4 Statistical analysis

Mean with standard deviation (SD) and median with 25th and 75th percentiles (interquartile range [IQR] boundaries) were used to present normally and skewed distributed continuous variables, respectively. The normality of the variables was assessed using histogram charts and central tendency and dispersion measures. Categorical variables were expressed as frequency and percentage. The Restricted Cubic Splines (RCS) in the Cox model allows a nonlinear relationship of lipid profile contents with the “ln hazard ratio” of mortality and MACCEs, estimated from the Cox regression model adjusted for all possible confounders. A cubic spline is a piecewise cubic polynomial, where the number of “pieces” is stated by the number of windows used. Inside each window is effectively a cubic polynomial, hence; these windows are expressed by “knots.” More specific details of the adjusted model (Model 1) (exp. Considered interaction terms) were discussed in Supporting Information. In brief, Model 1 adjusted for all possible confounders. dfmacox (degrees of freedom in multivariate additive Cox models) function in “SmoothHR package” were used to achieve the prime number of degrees of freedom in the adjusted multivariate model. The lowest risk point was considered as the reference value. Implementation of LDL‐C, HDL‐C, and remnant Cholesterol levels was plotted in two genders using hazard ratios (HR) and 95% confidence intervals (CI). Two main tests checked linearity and nonlinearity, Martingale residual plots for visual examination and ANOVA for statistical examination. The *p* < .1 considered as significant in mentioned tests. The Proportional hazard assumption was tested through a visual assessment based on the scaled Schoenfeld residuals for each variable. Statistical insignificance was set as *p* < .05 in a two‐tailed test. R programming language (R version 4.0.3) used for all statistical analyses.

## 3 RESULT

### 3.1 Clinical characteristics

In the final analysis, we included 17 555 patients who underwent isolated CABG procedures between January 2005 and December 2016. The median follow‐up was 75.24 [75.02, 75.58] months, equivalent to 99 004 person‐years of follow‐up. Baseline clinical data of the study patients are shown in Table 1. The mean age of the study population was 66 ± 9.98 years, and 72.8% (12778 patients) were male. The mean levels of HDL‐C, remnant cholesterol, and LDL‐C were 37.50 ± 9.13, 23.77 ± 11.74, and 94.37 ± 32.95, respectively.

**Table 1:** Baseline characteristics.

		Total cohort (*n* = 17 555)
Age (years)		66 ± 9.98
Gender	Female	27.2% (4777)
	Male	72.8% (12778)
BMI		27.29 ± 4.23
Diabetes		39.3% (6890)
Hypertension		54.5% (9560)
Dyslipidemia		55.2% (9693)
eGFR		80.62 ± 27.67
HDL‐C (mg/dL)		37.50 ± 9.13
Remnant cholesterol (mg/dL)		23.77 ± 11.74
LDL‐C (mg/dL)		94.37 ± 32.95
COPD		3.5% (615)
Cerebrovascular accident		NA
FH		36.7% (6440)
Opium consumption	No	85.7% (14960)
	Current	11.6% (2018)
	Former	2.8% (488)
Cigarette smoker	No	66.2% (11581)
	Current	17.1% (3001)
	Former	16.7% (2919)
Previous MI distance	No	83.6% (14609)
	≤24 h	3.2% (565)
	1–7 days	6.5% (1143)
	8–21 days	6.7% (1165)
	>21 days	NA
Status of procedure	Elective	95.6% (16742)
	Urgent or emergent	4.4% (775)
CPB utilization		91.4% (15609)
Ejection fraction (%)		50 [42, 55]
Graft number		3 [3, 4]
Statin		89.6% (12504)
Aspirin		73.5% (10071)
AT1 receptors antagonists		27.4% (3759)
Beta blockers		78.0% (11471)

### 3.2 Main outcomes

In Cox analysis, Quartile (Q3) (89 < LDL‐C < 113 and 26 < HDL‐C < 42) was served as the reference group. All analyses were reported separately in men and women.

− Among men, those in the highest LDL‐C level (Q4, LDL‐C > 114) and those in the lowest HDL‐C level (Q1, HDL‐C < 30) showed a significantly higher risk of all‐cause mortality compared to other groups (compared with Q3, LDL‐C Q4, HR = 1.16, 95% CI: 1.02–1.26, *p* = .014; HDL‐C Q1, HR = 1.14, 95% CI: 1.01–1.31, *p* = .041). In addition, male patients with the highest HDL‐C level (Q4, HDL‐C > 42) showed a significantly lower risk of all‐cause mortality compared to other groups (compared to Q3, HDL‐C Q4, HR = 0.92, 95% CI: 0.85–0.99, *p* = .029) (Table 2).
− Female patients in the lowest HDL‐C level (Q1, HDL‐C < 30) showed a significantly higher (compared with Q3, HR = 1.14, 95% CI: 1.01–1.31, *p* = .028) and those in the highest HDL‐C level (Q4, HDL‐C > 43) showed a significantly lower (compared with Q3, HR = 0.74, 95% CI: 0.58–0.98, *p* = .019) risk of all‐cause mortality (Table 3).

**Table 2:** Cox regression analyses of the relationship between lipid profile and mortality.

			Crude		Model 1	
			HR (95% CI)	*p* Value	HR (95% CI)	*p* Value
Male	LDL‐C concertation	Q1(<68 mg/dL)	1.10 (0.96–1.27)	.183	1.05 (0.91– 1.20)	.982
		Q2(69–88 mg/dL)	1.00 (0.88–1.16)	.959	0.98 (0.82– 1.18)	.872
		Q3(89–113 mg/dL)	1	Ref	1	Ref
		Q4(>114 mg/dL)	0.91 (0.79–1.04)	.170	1.16 (1.02– 1.26)	.014
	HDL‐C concertation	Q1(<30 mg/dL)	1.21 (1.06–1.38)	.004	1.14 (1.01– 1.31)	.041
		Q2(31–35 mg/dL)	1.11 (0.96–1.28)	.154	1.06 (0.92– 1.23)	.443
		Q3(36–42 mg/dL)	1	Ref	1	Ref
		Q4(>42 mg/dL)	1.01 (0.89–1.16	.839	0.92 (0.85–0.99)	.029
	Remnant cholesterol concertation	Continues	0.989 (0.985– 0.994)	<.001	1.002 (0.995– 1.013)	.229
Female	LDL‐C concertation	Q1(<75 mg/dL)	1.06 (0.92–1.23)	.421	1.19 (0.91–1.56)	.216
		Q2(76–96 mg/dL)	0.90 (0.78–1.05)	.178	0.94 (0.71– 1.25)	.941
		Q3(97–121 mg/dL)	1	Ref	1	Ref
		Q4(>122 mg/dL)	0.92 (0.86–1.15)	.923	1.14 (0.87–1.49)	.356
	HDL‐C concertation	Q1(<30 mg/dL)	1.27 (1.08–1.49)	.004	1.28 (1.02–1.69)	.028
		Q2(31–35 mg/dL)	1.03 (0.88–1.21)	.732	0.86 (0.65–1.15)	.865
		Q3(36–42 mg/dL)	1	Ref	1	Ref
		Q4(>43 mg/dL)	0.96 (0.85–1.11)	.564	0.74 (0.58–0.95)	.019
	Remnant cholesterol concertation	Continues	1.003 (0.997– 1.009)	.330	1.009 (1.003– 1.016)	.003

**Table 3:** Cox regression analyses of the relationship between lipid profile and ACS/revascularization.

			Model 1	
			HR (95% CI)	*p* Value
Male	LDL‐C concertation	Q1(<68 mg/dL)	0.89 (0.75– 1.05)	.176
		Q2(69–88 mg/dL)	0.93 (0.79– 1.10)	.872
		Q3(89–113 mg/dL)	1	Ref
		Q4(>114 mg/dL)	0.99 (0.84– 1.16)	.861
	HDL‐C concertation	Q1(<30 mg/dL)	1.06 (0.90– 1.24)	.505
		Q2(31–35 mg/dL)	1.09 (0.93– 1.28)	.443
		Q3(36–42 mg/dL)	1	Ref
		Q4(>42 mg/dL)	0.94 (0.80–1.11)	.445
	Remnant cholesterol concertation	Continues	1.004 (1.001– 1.008)	.031
Female	LDL‐C concertation	Q1(<75 mg/dL)	1.045 (0.82–1.33)	.696
		Q2(76–96 mg/dL)	0.92 (0.73– 1.15)	.455
		Q3(97–121 mg/dL)	1	Ref
		Q4(>122 mg/dL)	1.02 (0.83–1.25)	.869
	HDL‐C concertation	Q1(<30 mg/dL)	1.18 (0.92–1.53)	.191
		Q2(31–35 mg/dL)	1.02 (0.80–1.31)	.849
		Q3(36–42 mg/dL)	1	Ref
		Q4(>43 mg/dL)	1.05 (0.86–1.28)	.607
	Remnant cholesterol concertation	Continues	1.005 (1.002– 1.012)	.008

**Figure 1:**
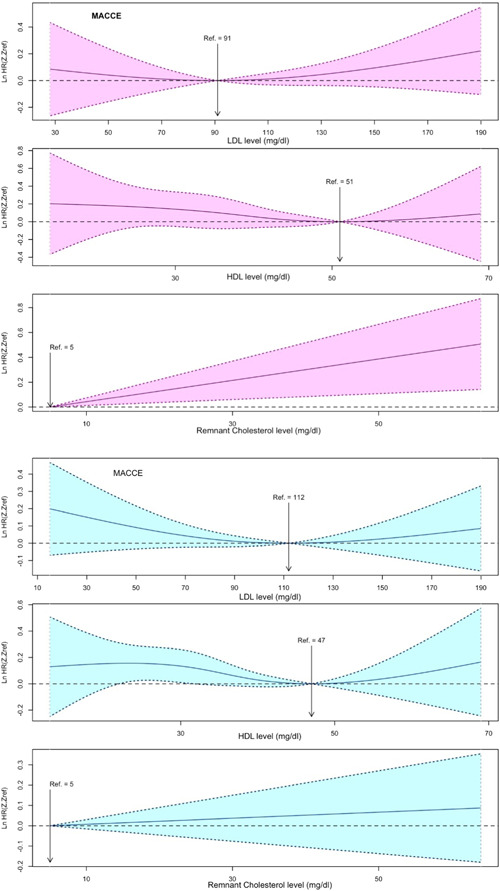
Restricted cubic spline cox regression analysis of lipid indices and risk of MACCE in women (pink) and men (blue).

**Figure 2:**
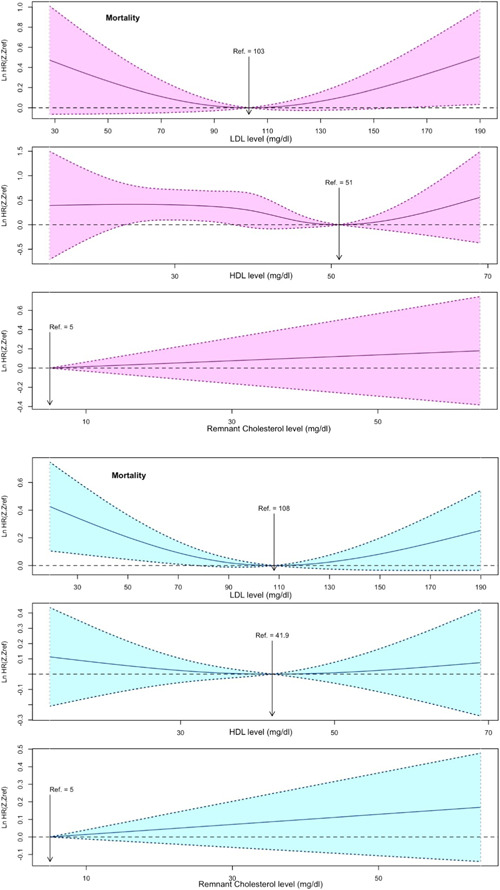
Restricted cubic spline cox regression analysis of lipid indices and risk of mortality in women (pink) and men (blue).

Multivariable cox models of RCS of lipid indices and MACCEs and mortality are shown in Figures 1 and 2, respectively. The relationship between LDL‐C and HDL‐C with all‐cause mortality was nonlinear (nonlinear *p* were .004 and <.001, respectively). In women, the lowest risk of MACCE and all‐cause mortality was located at LDL‐C concentration of 91 and 103 and at HDL‐C concentration of 51 (for both MACCE and mortality); while in men, the lowest risk of MACCE and all‐cause mortality was located at LDL‐C concentration of 112 and 108, and at HDL‐C concentration of 47 and 41.9, respectively. Both extremely high and extremely low levels of LDL‐C was associated with a high risk of MACCE and mortality. Moreover, extremely high levels of HDL‐C were associated with a high risk of post‐CABG adverse outcomes.

The relationship between remnant cholesterol and all‐cause mortality was linear (linearity *p* = .023). In female patients, each 1 unit increase in remnant cholesterol had significant but week associated with a higher risk of mortality (HR = 1.009, 95% CI: 1.003–1.016, *p* = .003); however, this association was not significant in male patients (*p* = .229).

### 3.3 Subgroup analysis

Among both males and females, those in the lowest LDL‐C level (Q4, LDL‐C > 114) and those in the highest HDL‐C level (Q1, HDL‐C < 30) showed a lower risk of ACS and revascularization compared to other groups.

## 4 DISCUSSION

The present study showed that, as continuous variables, there was a nonlinear relationship between LDL‐C and HDL‐C with post‐CABG adverse events. This means both extraordinarily high and low HDL‐C and LDL‐C concentrations were associated with high mortality risk and MACCE. In women, LDL‐C of 103 and HDL‐C of 51 and in men, LDL‐C of 108 and HDL‐C of 42 was associated with the lowest mortality risk. However, when presented as categorical variables, higher levels of HDL‐C were associated with a lower risk of mortality in both women and men. As a continuous variable, remnant cholesterol was positively associated with the risk of post‐CABG mortality in women.

### 4.1 HDL‐C and LDL‐C

Previous studies suggested an inverse and linear relationship between HDL‐C and LDL‐C levels and overall mortality in patients with cardiovascular disease^14^, ^15^, ^16^; however, more recently published studies have observed a nonlinear relationship. For instance, Mendelian randomization studies have shown that increased HDL‐C levels caused by common variants in HDL‐related genes are not necessarily associated with a lower incidence of cardiovascular events.^22^, ^23^ Several large‐scale cohort studies also disproved the previous finding of a linear inverse relationship between HDL‐C and cardiovascular disease.^17^, ^18^, ^19^, ^20^ There is no specific goal determined for HDL and LDL and treatment may vary among individuals.^24^ Followingly, ESC 2021 stated that very high HDL‐C might signal increased cardiovascular risk and that there have not yet been clinical trials of cholesteryl ester transfer protein inhibitors from which a therapeutic goal for HDL‐C can be achieved.^22^, ^25^ One possible explanation for the association between high HDL‐C and higher post‐CABG adverse outcomes is that high HDL‐C concentrations often are due to genetic variants,^26^ which may have unfavorable effects causing a high risk of disease and death.^27^, ^28^ Another explanation is that HDL‐C in patients with CAD or ACS loses endothelial anti‐inflammation capacity and cannot induce endothelial repair due to a lack of endothelial nitric oxide stimulation.^29^ As shown in this study, considering HDL‐C as a categorical variable may fail to clarify inverse associations at extremely high concentrations and thus, may underestimate the cardiovascular risk of individuals with very high HDL‐C values.

Moreover, several studies have shown that low levels of LDL‐C are associated with increased risk of all‐cause mortality and cardiovascular disease mortality.^30^, ^31^, ^32^ These findings, along with the nonlinear relationship between LDL‐C and post‐CABG adverse outcomes in our study, provide evidence supporting the “lipid paradox,” suggesting that lower cholesterol concentrations do not always confer protective effects on cardiovascular outcomes. While the exact mechanisms of this paradox remain unclear, one possible explanation is that a low LDL‐C concentration is shown to be associated with a fatal disease. Some studies have shown that LDL‐C inactivates a broad range of microorganisms and toxic products, which might be a possible causal factor of cardiovascular disease and cancer.^33^, ^34^ Another explanation could be the higher prevalence of malnutrition and its potential side effects in patients with low levels of LDL‐C.^35^ Moreover, patients with baseline high LDL‐C concentrations are more likely to receive post‐CABG high dose lipid‐lowering treatments, which may contribute to a lower risk of adverse outcomes. However, this study was not intended to evaluate the impact of treatment interventions on lipid levels and outcomes, and the results were adjusted for preadmission lipid‐lowering treatments.

To the best of our knowledge, no previous study has examined the concentration of lipid indices associated with the lowest risk of post‐CABG adverse outcomes; however, some studies evaluated the risk of cardiovascular disease and death in the general population. Dong et al.^21^ evaluated the effect of lipid indices on the risk of cardiovascular disease (CVD), all‐cause death, and CVD death during the 10‐year follow‐up using RCS analysis. Similar to our findings, they showed that HDL‐C had a nonlinear relationship with CVD and CVD death, while remnant cholesterol had a linear relationship with CVD death. They presented cut‐off points of lipid indices for each outcome based on RCS and found that the median cut‐off point for HDL‐C was 51, LDL‐C was 102, and remnant cholesterol was 29 mg/dL. Another study conducted by Johannesen et al.^31^ evaluated the association between levels of LDL‐C and cardiovascular mortality, and the concentration of LDL‐C associated with the lowest risk of cardiovascular mortality in the general population, using RCS analysis. They showed a nonlinear relationship between LDL‐C and cardiovascular mortality, and the lowest risk of cardiovascular mortality was at an LDL‐C concentration of 132 mg/dL. Our study found that the lowest risk of post‐CABG MACCEs was at LDL‐C concentration of 91 in women and 112 in men and HDL‐C concentration of 51 in women and 47 in men. Our findings regarding the gender differences in HDL‐C optimal cut off are in line with the fact that different cut‐off values for men and women are used in the National Cholesterol Education Program (NCEP) criteria for metabolic syndrome, which is higher in women than in men.^36^

### 4.2 Remnant cholesterol

Remnant cholesterol is the cholesterol content of triglyceride‐rich lipoproteins, which is also referred to as triglyceride‐rich lipoprotein cholesterol. Remnant cholesterol is composed of very‐low‐density lipoproteins and intermediate‐density lipoproteins in the fasting state and chylomicron remnants in the nonfasting state.^37^ Remnant cholesterol is more likely to get trapped in the intima than LDL‐C because of its larger size and more efficiently is taken up by macrophages than LDL‐C, which leads to a faster formation of foam cells.^38^ As a single marker, the use of LDL‐C in predicting adverse cardiovascular outcomes could be challenged as the magnitude of LDL‐C reduction does not reflect clinical outcomes in patients using intensive lipid‐lowering therapy. Moreover, despite reducing LDL‐C to recommended levels, there is still a considerable residual risk of adverse cardiovascular outcomes.^39^ Recent data suggested that remnant cholesterol is associated with cardiovascular outcomes, independent of other risk factors, including LDL‐C and thus, is contributed to this residual risk.^40^, ^41^ The increased risk of adverse cardiovascular outcomes associated with remnant cholesterol could also be attributed to mechanisms related not only to atherosclerotic plaque formation but also to local inflammation, by inducing the production of cytokines (tumor necrosis factor‐α), interleukins (IL‐1, IL‐6, IL‐8), and pro‐atherogenic adhesion molecules activating inflammation.^42^ Castaner et al.^40^ evaluated the association between remnant cholesterol and major cardiovascular events in a cohort of older individuals at high cardiovascular risk. They showed that individuals with remnant cholesterol levels ≥30 mg/dL had a higher risk of major cardiovascular events, regardless of whether LDL‐C was at optimal levels or not. Another study conducted by Cao et al.^43^ examined the prognostic value of remnant cholesterol in the patients with CAD and showed that high remnant cholesterol levels as categorical and continuous variables were independent risk factors for major cardiovascular events. Another study conducted by Kugiyama et al.^44^ showed that higher remnant cholesterol levels predict future coronary events in patients with CAD independently of other risk factors. In our study, we found that there is a positive linear relationship between remnant cholesterol and post‐CABG adverse outcomes in both women and men.

### 4.3 Strength and limitation

Our study has several important strengths, including large sample size and a relatively long follow‐up period. All LDL‐C levels were measured directly in this study, which should be considered a significant strength. Moreover, our study explored the association between lipid indices and post‐CABG outcomes by applying both cox regression and RCS methods to show the possible nonlinear relationship. However, several limitations should be acknowledged. At first, this conducted study was conducted in a single center. However, THC is a referral educational university that serves patients from all over the country. Second, we did not have permission to have the “autopsy” report of the patients; hence, the cause of death was unclear. Third, relevant risk factors (such as lifestyle and malignancy) may have confounding effects on long‐term mortality.

## 5 CONCLUSION

In this large cohort study, we showed a nonlinear relationship between HDL‐C and LDL‐C and post‐CABG adverse outcomes, in which extremely low or high levels of HDL‐C and LDL‐C were associated with higher risk MACCE and all‐cause mortality. However, there was a linear relationship between remnant cholesterol and adverse outcomes.

## AUTHOR CONTRIBUTIONS

Ali Sheikhy and Aida Fallahzadeh contributed in drafting. Ali Sheikhy contributed in formal analysis. Saeed Sadeghian contributed in scientific supervision. Mina Pashang contributed in data gathering and cleaning. Abbas Ali Karimi, Jamshid Bagheri, and Hossein Ahmadi‐Tafti contributed in data gathering. Kaveh Hosseini contributed in correspondence, final editing, and supervision.

## CONFLICT OF INTEREST STATEMENT

The authors declare no conflict of interest.

## Supporting information

Supporting information.

## Data Availability

The data that support the findings of this study are available from the corresponding author upon reasonable request. The data underlying this article will be shared on reasonable request to the corresponding author.
