# Supplementary material for: A realistic approach to evaluating the effect of baseline lipid profile in postcoronary artery bypass grafting surgery
Source: Clin Cardiol. 2023 Aug 18;46(11):1408–17. doi: 10.1002/clc.24132 (PMC10642323; doi:10.1002/clc.24132)
Supplement: Supplementary file 1 — Supporting information. [file CLC-46-1408-s001.docx]

**Outcome**

Survival status was determined using the local region’s electronic registry on the 31st December 2016.

The “time” defined as days from discharge to death or censoring at study endpoint, whichever came first. The median follow up was 75.24 [75.02, 75.58] months, which measured by reverse Kaplan-Meier method. Figure Supp 1 demonstrates baseline survival function.


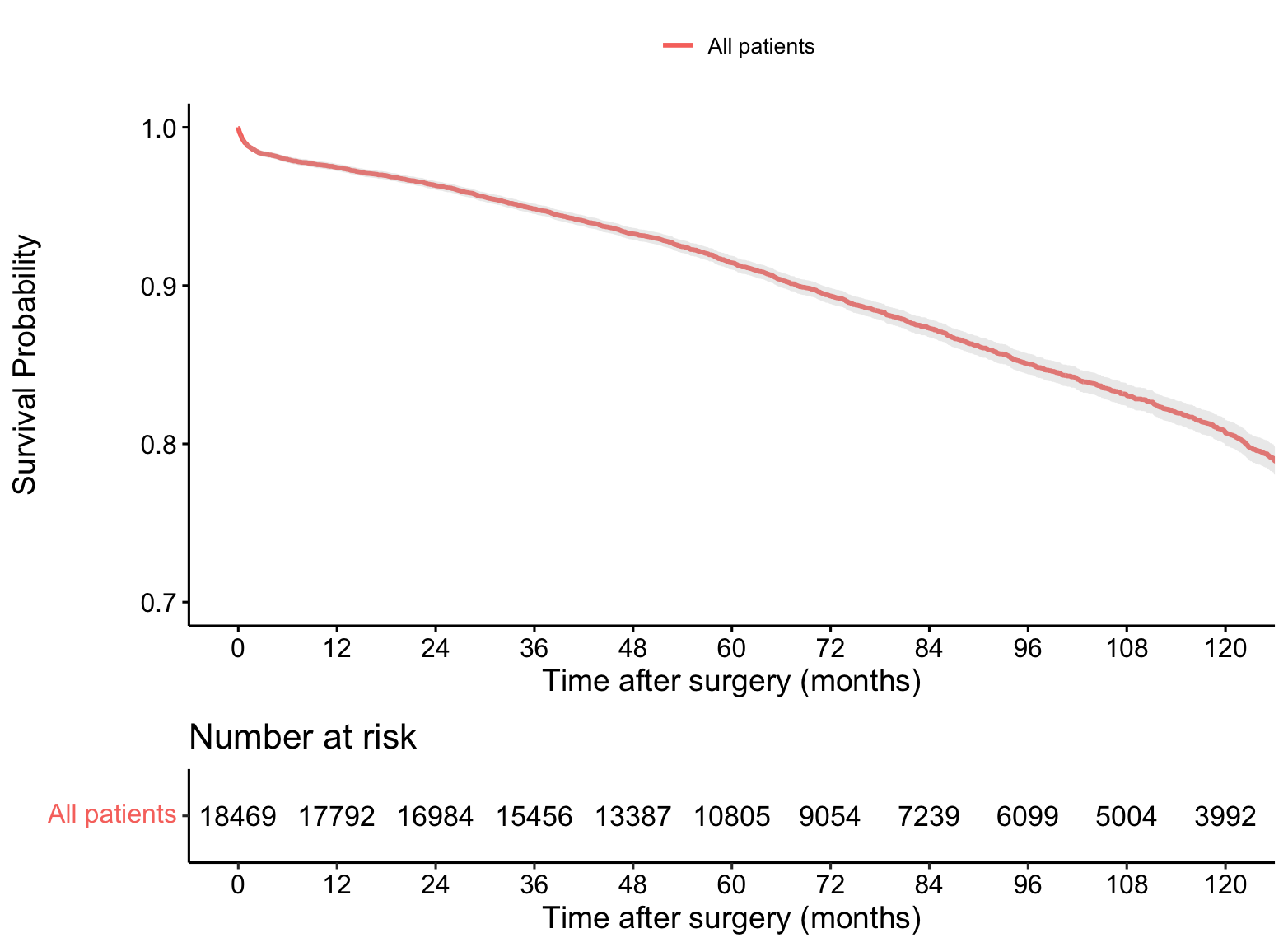


figure Supp 1. Baseline survival function.

All patients with missing survival status excluded from the study.

**Variable considerations**

**Missing variables**

Complete interpretation of missing variables is shown graphically in figures e2 and 3.


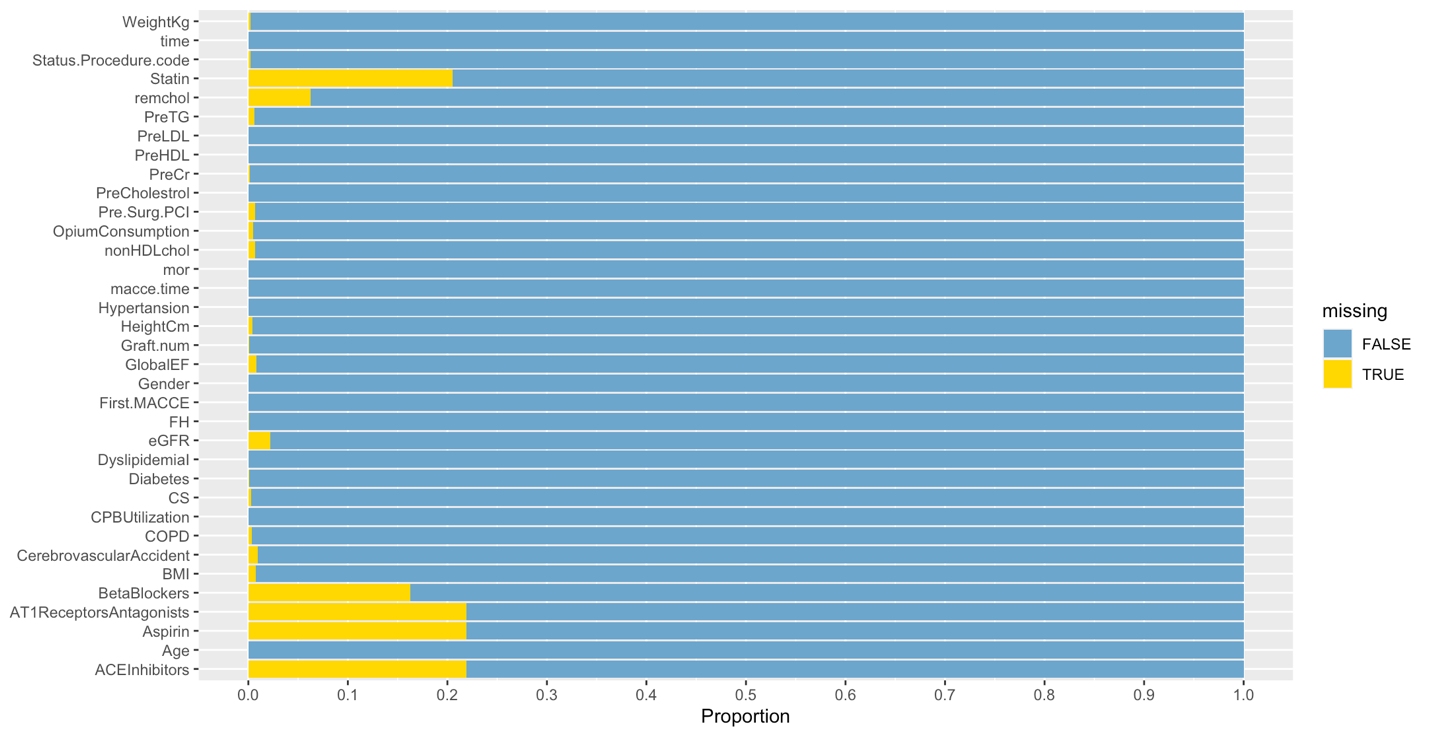


Fig e2. Missing proportion of each variable


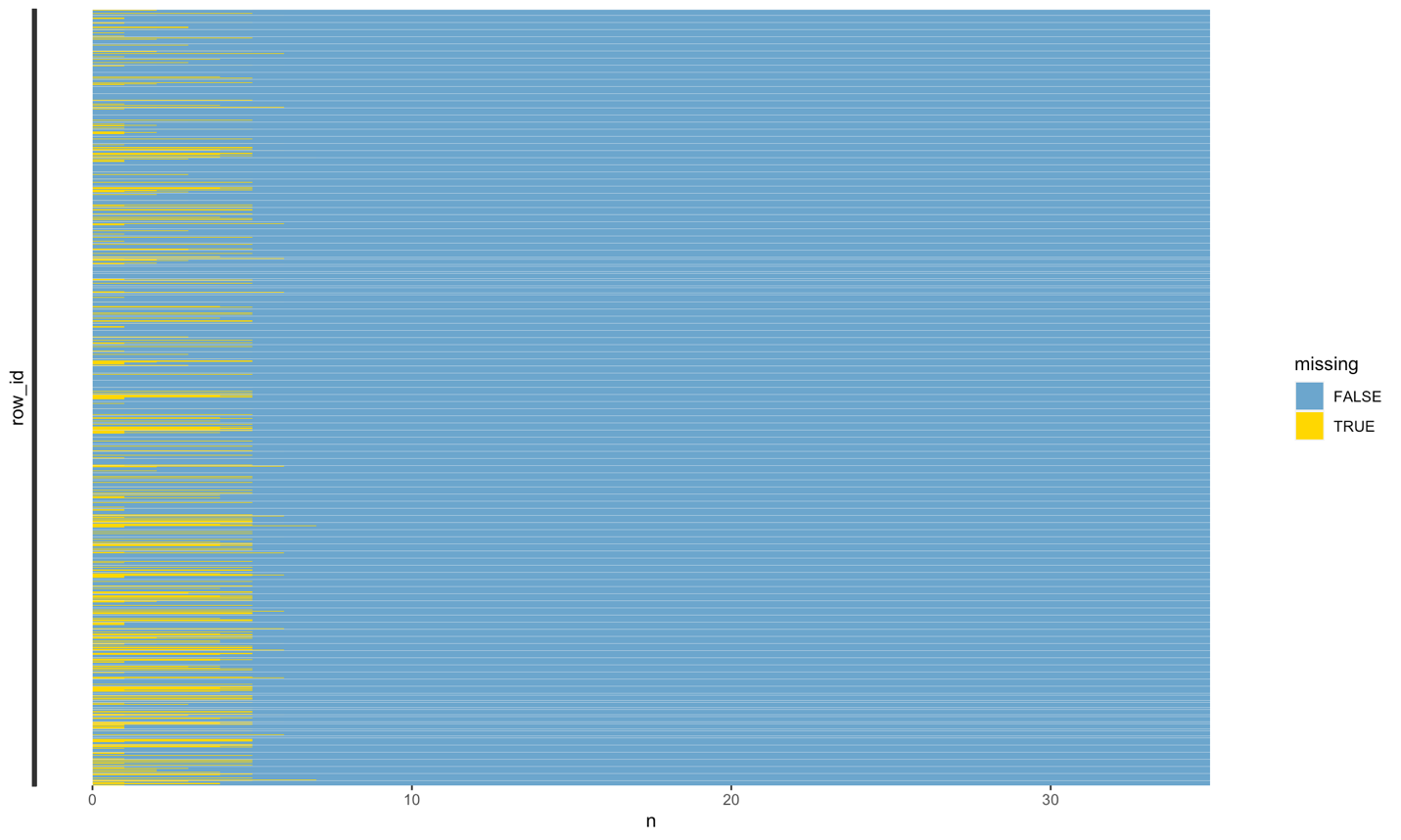


Fig e3. Number of missed variables in each case

**Variable definitions**

eGFR was measured with the Cockcroft-Gault formula ((140-age) * weight * constant)/Serum Creatinine in μmol/L. moreover we did not use calculated LDL, all reported LDL levels were measured directly at Tehran Heart Center (THC) laboratory. Used method is described below:


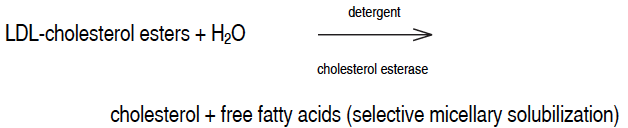


Cholesterol esters are broken down quantitatively into free cholesterol and fatty acids by cholesterol esterase.


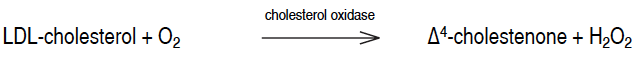


In the presence of oxygen, cholesterol is oxidized by cholesterol oxidase to Δ^4^‑cholestenone and hydrogen peroxide.


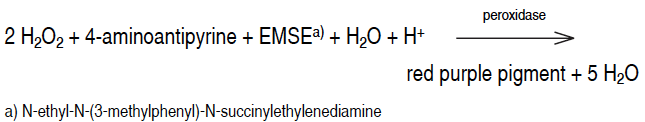


In the presence of peroxidase, the hydrogen peroxide generated reacts with 4‑aminoantipyrine and EMSE to form a red purple dye. The color intensity of this dye is directly proportional to the cholesterol concentration and is measured photometrically.

Diabetes was defined as fasting plasma glucose ≥ 126 mg/dL or random plasma glucose ≥ 200

mg/dL and/or hemoglobin A1c (HbA1c) ≥ 6.5% and/or treatment with either oral hypoglycemic agents or insulin. Hypertension was defined as a minimum systolic blood pressure of 140 mm Hg or minimum diastolic blood pressure of 90 mm Hg or a history of receiving antihypertensive medication. Dyslipidemia was defined as the presence of a minimum total cholesterol level of 240 mg/dL, a minimum triglyceride level of 200 mg/dL, a high-density lipoprotein cholesterol level of less than 40 mg/dL in men and less than 50 mg/dL in women, a minimum low-density lipoprotein cholesterol level of 160 mg/dL, or a history of prescribed lipid-lowering drugs based on the National Cholesterol Education Program (NCEP) Adult Treatment Plan (ATP) III. A family history of coronary artery disease (CAD) was defined as having a first-degree relative with a history of CAD, including acute myocardial infarction or documented CAD (through invasive coronary angiography or computed tomography coronary angiography). Cigarette smoking and opium consumption were determined according to the patient’s self-reported status. A current smoker was defined as an individual who has smoked more than 100 cigarettes and currently smokes. Opium consumption was defined as the current consumption of opium, whichever smoking opium or drinking opium dissolved in tea.

**Extreme Outliers**

*Outliers* are defined as observations with more than three interquartile ranges over the third quartile or below the first quartile, which these extreme values may affect a regression

model significantly. In this study, data screening was performed, outliers excluded, and the boxplot of figure e4 demonstrates the final results.

| 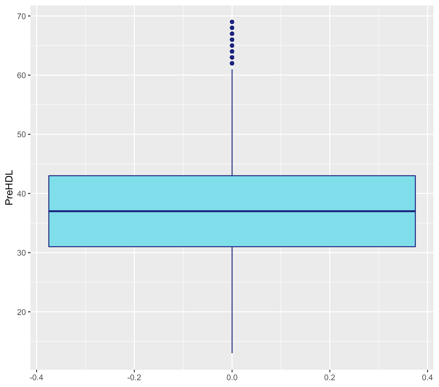 | 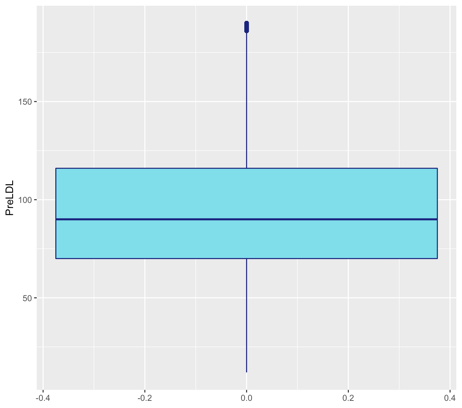 | 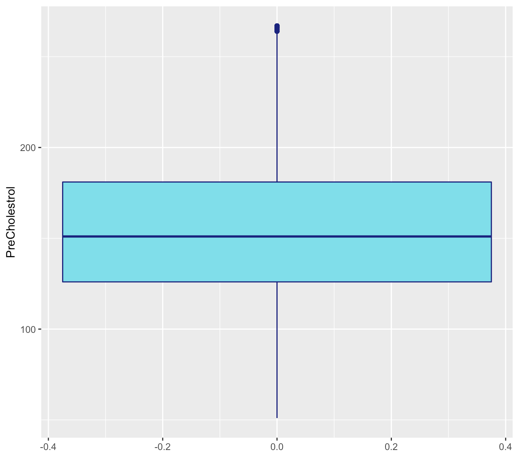 |
| --- | --- | --- |
| HDL | LDL | Cholesterol |

| 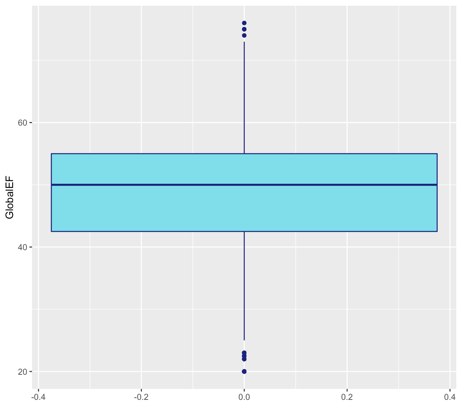 | 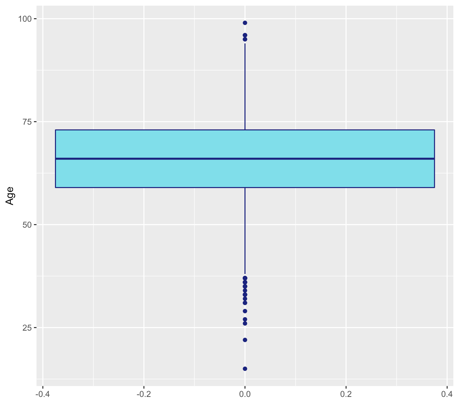 | 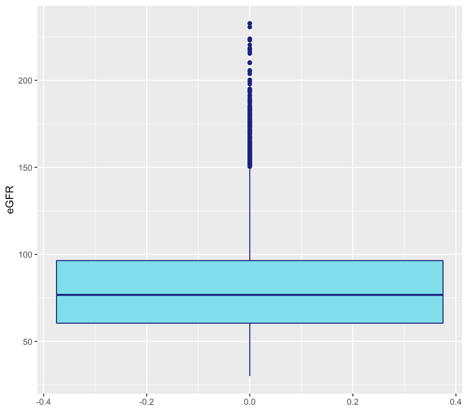 |
| --- | --- | --- |
| EF | Age | eGFR |

figure e4. Boxplots of the continuous predictors.

**Non-Linear effect**

All continues variables were tested for non-linearity by using 3-knot splines followed by ANOVA tests to determine a significant non-linear component. LDL, HDL, eGFR and EF showed a non-linear effect; hence, age and remnant cholesterol were linear.

LDL P-non linearity=0.004

HDL P-non linearity<0.001

eGFR P-non linearity<0.001

EF P-non linearity<0.001

Remnant cholesterol P-linearity = 0.023, Remnant cholesterol P-non linearity = 0.834 (Figure e5)


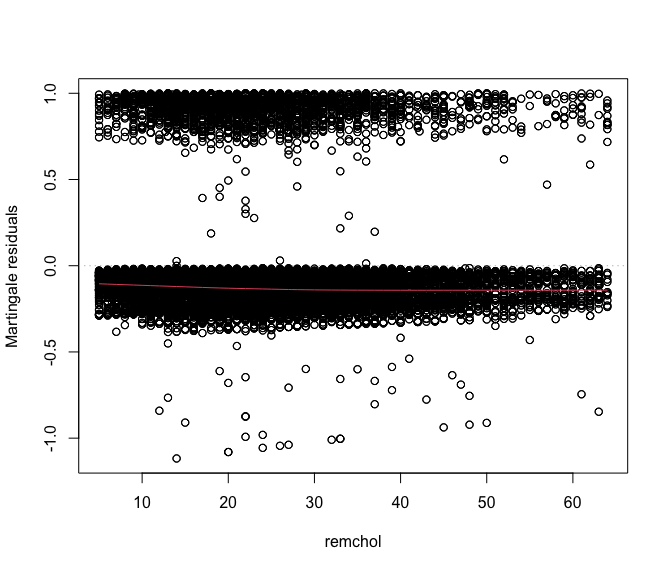


Fig e5. Graphical test for Remnant cholesterol linearity

**Number of Knots selection**

To select optimal number of knots (df +1), to reach statistical balancing act between bias and variance, we used *smoothHR* package via mentioned function below:

df1 <- dfmacox (time= "time", status = "mortality", nl.predictors = c ("PreLDL"), smoother = "ns", method = "AIC", data = data)

df(PreLDL) = 3

df(PreHDL) = 3

df(remnant_cholesterol) = 1

**Fitting the multivariate models**

The fitted model was adjusted by covariates such as age, BMI, eGFR, Diabetes, Hypertension, Cigarette smoking, Ejection Fraction, Graft number, anti-lipid and anti-hypertensive medications.

To overcome the two-way interaction confounders, we tested and adjusted only clinically plausible interactions to avoid overfitting. We considered interaction terms of sex * BMI, HDL*BMI, LDL*BMI, and Hypertension*eGFR in our model. three-way interactions (exp. HDL*BMI*Anti_hypertensive_drugs) were omitted in this study.

**Proportional hazards assumption**

Graphically plot and Schoenfeld residuals used to test PH assumption. The global P-value for our model was 0.84 in mortality as the outcome and 0.73 in MACCE as the outcome. Accordingly, there was no sign of violation of PH assumption.

PH assumptions were tested for each variable separately. None of the variables showed a violation either.
